# Impact of excluding nursing home COVID-19 cases when assessing the relationship between county-level social distancing behavior and COVID-19 cases across the US during the early phase of the pandemic, February 2020-May 2020

**DOI:** 10.1371/journal.pone.0260151

**Published:** 2021-11-30

**Authors:** Phoebe Tran, Lam Tran, Liem Tran

**Affiliations:** 1 Department of Chronic Disease Epidemiology, Yale University, New Haven, CT, United States of America; 2 Department of Biostatistics, Michigan School of Public Health, Ann Arbor, MI, United States of America; 3 Deparment of Geography, University of Tennessee, Knoxville, TN, United States of America; Nanyang Technological University, SINGAPORE

## Abstract

**Objectives:**

To conduct a cross-sectional nationwide study examining how exclusion of nursing home COVID-19 cases influences the association between county level social distancing behavior and COVID-19 cases throughout the US during the early phase of the pandemic (February 2020-May 2020).

**Methods:**

Using county-level COVID-19 data and social distancing metrics from tracked mobile devices, we investigated the impact social distancing had on a county’s total COVID-19 cases (cases/100,000 people) between when the first COVID-19 case was confirmed in a county and May 31^st^, 2020 when most statewide social distancing measures were lifted, representing the pandemic’s exponential growth phase. We created a mixed-effects negative binomial model to assess how implementation of social distancing measures when they were most stringent (March 2020-May 2020) influenced total COVID-19 cases while controlling for social distancing and COVID-19 related covariates in two scenarios: (1) when COVID-19 nursing home cases are not excluded from total COVID-19 cases and (2) when these cases are excluded. Model findings were compared to those from February 2020, a baseline when social distancing measures were not in place.

Marginal effects at the means were generated to further isolate the influence of social distancing on COVID-19 from other factors and determine total COVID-19 cases during March 2020-May 2020 for the two scenarios.

**Results:**

Regardless of whether nursing home COVID-19 cases were excluded from total COVID-19 cases, a 1% increase in average % of mobile devices leaving home was significantly associated with a 5% increase in a county’s total COVID-19 cases between March 2020-May 2020 and about a 2.5% decrease in February 2020. When the influence of social distancing was separated from other factors, the estimated total COVID-19 cases/100,000 people was comparable throughout the range of social distancing values (25%-45% of mobile phone devices leaving home between March 2020-May 2020) when nursing home COVID-19 cases were not excluded (25% of mobile phones leaving home: 163.84 cases/100,000 people (95% CI: 121.81, 205.86), 45% of mobile phones leaving home: 432.79 cases/100,000 people (95% CI: 256.91, 608.66)) and when they were excluded (25% of mobile phones leaving home: 149.58 cases/100,000 people (95% CI: 111.90, 187.26), 45% of mobile phones leaving home: 405.38 cases/100,000 people (95% CI: 243.14, 567.62)).

**Conclusions:**

Exclusion of nursing home COVID-19 cases from total COVID-19 case counts has little impact when estimating the relationship between county-level social distancing and preventing COVID-19 cases with additional research needed to see whether this finding is also observed for COVID-19 growth rates and mortality.

## Introduction

During the first half of 2020, the COVID-19 pandemic rapidly emerged as a serious and ongoing public health threat in the US [1,2]. Between March 1^st^ and May 31^st^, 2020, the implementation of statewide social distancing measures such as sheltering in place/stay at home orders, closing stores, forbidding large groups from gathering, and requiring a distance of six feet between individuals played a critical role in reducing the number of COVID-19 cases [3–5]. Yet, despite the importance of social distancing in COVID-19 prevention, following these measures proved difficult for the 1.3 million Americans who live in nursing homes [6,7].

Recognizing social distancing behavior can vary within a state even with statewide social distancing measures in place, several nationwide US studies have assessed the impact of social distancing on COVID-19 at the county level [8–12]. However, these studies do not separate out nursing home COVID-19 cases from total COVID-19 cases in their analyses [8–12]. This action may obscure the actual impact of county-level social distancing behavior in preventing COVID-19 as the social distancing behavior of nursing home residents is atypical of the general US population [8–12]. Thus, we sought to assess how the exclusion of nursing home COVID-19 cases influences the relationship between county-level social distancing behavior and COVID-19 cases throughout the US during the early phase of the pandemic when COVID was spreading exponentially.

## Methods

### Data sources

#### Ethics

All study data is anonymized and publicly available online. Prior informed consent and IRB approval have been obtained by the institutions (Johns Hopkins University, SafeGraph) and government agencies (Centers for Disease Control and Prevention, Centers for Medicare & Medicaid Services) providing this data before data release [13,14,17,21]. As a result, we are exempt from needing to seek further informed consent and IRB approval from our respective institutions." [13,14,17,21].

#### Total COVID-19 confirmed cases

We retrieved COVID-19 confirmed case counts for all 3,142 US counties from the Johns Hopkins University, Center for Systems Science and Engineering Coronavirus Resource Center (https://systems.jhu.edu) [13]. Data on COVID-19 cases in this dataset come from the US Centers for Disease Control and Prevention (CDC) and state health departments, making it one of the most comprehensive sources of information on county-level confirmed COVID-19 cases available in the US [13]. For each county, we defined total confirmed COVID-19 cases (cases/100,000 people) to be the cumulative number of confirmed COVID-19 cases starting from when the state a county is in had its first confirmed COVID-19 case and up to May 31^st^, 2020 when the majority of statewide social distancing measures were lifted [4]. A county’s total confirmed COVID-19 cases was calculated using the following equation:

$$Total\;confirmed\;COVID–19\;cases=\frac{county^{\prime }s\;confimed\;COVID-19\;cases}{county^{\prime }s\;population}*100,000.$$

We chose to model COVID-19 cases rather than deaths because geographic differences in hospital specific factors (i.e., number of ICU beds, average ventilator use) may have a larger impact on COVID-19 mortality than social distancing behavior. In addition, we focused on these specific first few months of the pandemic as a more expanded period would introduce more factors which are hard to control for in the analysis such as changes in case reporting protocols by states at different times and social distancing behavior (i.e., large gatherings, indoor dining) varying widely from state to state that cannot be easily captured by existing data once stay-at-home orders were lifted.

#### Nursing home COVID-19 cases

Information on nursing home COVID-19 cases within the same time period was obtained from the Centers for Medicare and Medicaid Services (CMS) (https://data.cms.gov/stories/s/COVID-19-Nursing-Home-Data/bkwz-xpvg/) [14]. For every US county, we used ArcGIS Pro 2.5 to determine all nursing home COVID-19 cases in facilities within a county’s geographic boundaries [15]. Nursing home COVID-19 cases were subtracted from a county’s total confirmed COVID-19 cases in estimates that excluded nursing home COVID-19 cases.

#### Social distancing metrics

Social distancing in terms of stay-at-home orders/sheltering in place was assessed through two metrics, average % of mobile devices leaving home between March 2020-May 2020 and the average % of mobile devices leaving home in February 2020. The average % of mobile devices leaving home between March 2020-May 2020 represents the impact of social distancing on COVID-19 cases when social distancing measures in the US were most stringent [3–5]. We use the average % of devices leaving home in February 2020 as a baseline to compare with the average % of mobile devices leaving home between March 2020-May 2020 as February 2020 represents when COVID-19 began to quickly spread across the US [16]. Information on these social distancing metrics was gathered from the SafeGraph COVID-19 Consortium (https://www.safegraph.com/academics) which collects social distancing data through GPS tracking of mobile phone devices [17].

#### Covariates

We identified factors associated with social distancing and COVID-19 from prior literature in these areas to include as study covariates [18–20]. Covariates included in our study were days between when the first confirmed COVID-19 case was reported in a county and May 31^st^, 2020, population density, and social vulnerability. We identified the days between when the first confirmed case was reported and May 31^st^, 2020 for each county from Johns Hopkins University’s COVID-19 dataset [13]. The population density of a county (persons per square mile) was calculated using data on population and county size from the United States Census Bureau [21]. Information on social vulnerability for each county was based off the CDC’s Social Vulnerability Index (https://www.atsdr.cdc.gov/placeandhealth/svi/index.html) and consists of 15 social vulnerability metrics (% of population below poverty, unemployment rate, per-capita income, % of population >25 years with no high school diploma, % of population >65 years, % of population <17 years, % of civilian non-institutionalized population with a disability, % of population that is a single parent household with children <18 years, % of population that is a minority, % of population >5 years who speak English “less than well”, % of housing that is a structure with >10 units, % of housing that is a mobile home, % of occupied housing units with more people than rooms, % of households with no vehicles, % of population in institutionalized group quarters) [22].

### Statistical analyses

#### Spatial autocorrelation testing and modelling

As spatial autocorrelation may be present in the data, we used Moran’s I to test for this [23]. The Moran’s I value was 0.21 (p-value <0.0001) for total confirmed COVID-19 cases but the Moran’s I of the regression errors significantly decreased to 0.12 (p-value <0.0001) when the outcome was modelled using a mixed-effects negative binomial regression showing spatial autocorrelation to be minimal when handled in this manner [23]. Thus, we used mixed-effects negative binomial models to examine the relationship between county-level social distancing behavior and a county’s total confirmed COVID-19 cases because we are modeling a rate (cases/100,000 people) where we expect to see a large amount of variability across different states and want to minimize spatial autocorrelation.

The influence of county-level social distancing behavior on a county’s total confirmed COVID-19 cases was modelled under two scenarios: (1) when nursing home COVID-19 cases are not excluded from total confirmed COVID-19 cases and (2) when nursing home COVID-19 cases are excluded. In the first scenario, total confirmed COVID-19 cases within a county was set as the model outcome with social distancing metrics as the exposure and previously mentioned covariates included for adjustment purposes. For the second scenario, model exposure and covariates remained the same while the outcome was the number of confirmed COVID-19 cases within a county when nursing home COVID-19 cases are subtracted from a county’s total confirmed COVID-19 cases. Additionally, we included a random intercept by state in the models to deal with correlation that could potentially arise from counties within the same state having similar behavioral factors, healthcare systems, and COVID-19 response and testing policies.

Model estimates for the two scenarios were generated for county level social distancing between March 2020-May 2020 when stay-at-home orders were in place and as a comparison for county level social distancing in February 2020 when stay-at-home orders were not implemented. We reported incidence rate ratios (IRRs) from the mixed-effects negative binomial models by exponentiating model coefficients. Moran’s I was conducted in ArcGIS Pro 2.5 using the Spatial Autocorrelation (Global Moran’s I) tool and modelling in Stata 17 with the *menbreg* command (see S1 Appendix for further detail). Statistical significance during spatial autocorrelation testing and mixed-effects negative binomial modelling were assessed using two-sided tests at α = 0.05 [15,24].

#### Marginal effects at the mean analysis

Using the results from our mixed-effects negative binomial model, we determined how social distancing in isolation from other covariates influences the number of total confirmed COVID-19 cases up to May 31^st^, 2020 using marginal effects at the mean for the two scenarios outlined above. Marginal effects at the mean are calculated by setting all values besides the variable of interest to the average value of that particular covariate [25]. For instance, this means that for each county the variables for social distancing retain their actual values while for all counties the variables for days between when the first confirmed case was reported and May 31^st^, 2020, population density, and social vulnerability would all be set to these covariates’ respective average value [25]. This technique allows us to more clearly separate the impact of social distancing from other factors on COVID-19 cases compared to simply obtaining estimates directly from a regression model [25]. We plotted the number of total confirmed COVID-19 cases up to May 31^st^, 2020 against the range of county-level social distancing (average % of mobile devices leaving home between March 2020-May 2020) values (25%-45%). Statistical significance was assessed using two-sided tests conducted at α = 0.05. Marginal effects at the mean analysis was performed with Stata 17 statistical software (*margins* command with the *at means* option) while data plotting was carried out in R Version 4.0 [24,26].

## Results

Our study included 1,771,243 total confirmed COVID-19 cases with 8.0% of cases being nursing home COVID-19 cases (Table 1). Findings from the mixed-effects negative binomial model show a significant association (p-value <0.001) between the average % of mobile devices leaving home between March 2020-May 2020 and a county’s total COVID-19 confirmed cases under the two scenarios. The IRR for average % of mobile devices leaving home between March 2020-May 2020 was found to be 1.050 (95% CI: 1.024, 1.077) when nursing home COVID-19 cases were not excluded and 1.051 (95% CI: 1.025, 1.078) when these cases were excluded, meaning that total COVID-19 confirmed cases in a county would be predicted to increase by 5.0% and 5.1% respectively for each 1% rise in the average % of mobile devices leaving home between March 2020-May 2020. Looking at average % of mobile devices leaving the home in February 2020, we see the opposite occurring with each 1% increase in the average % mobile devices leaving the home associated with a 2.5% decrease in total COVID-19 confirmed cases when nursing home COVID-19 cases were not excluded and a 2.6% decrease in total COVID-19 confirmed cases when nursing home COVID-19 cases are excluded. Additional details on model parameters and goodness of fit can be found in S1 Table.

**Table 1:** Mixed-effects negative binomial model assessing influence of social distancing behavior on US COVID-19 cases (cases/100,000 people) between when a county had its first confirmed case and May 31^st^, 2020.

Variables	Not excluding nursing home COVID-19 cases (n = 1,771,243)			Excluding nursing home COVID-19 cases (n = 1,629,827)		
	Incidence Rate Ratio	95% Confidence Interval	P value	Incidence Rate Ratio	95% Confidence Interval	P value
Average % of mobile devices leaving home between March 2020-May 2020	1.050	(1.024, 1.077)	<0.001	1.051	(1.025, 1.078)	<0.001
Average % of mobile devices leaving the home in February 2020	0.975	(0.953, 0.997)	<0.001	0.974	(0.952, 0.996)	<0.001

**Fig 1:**
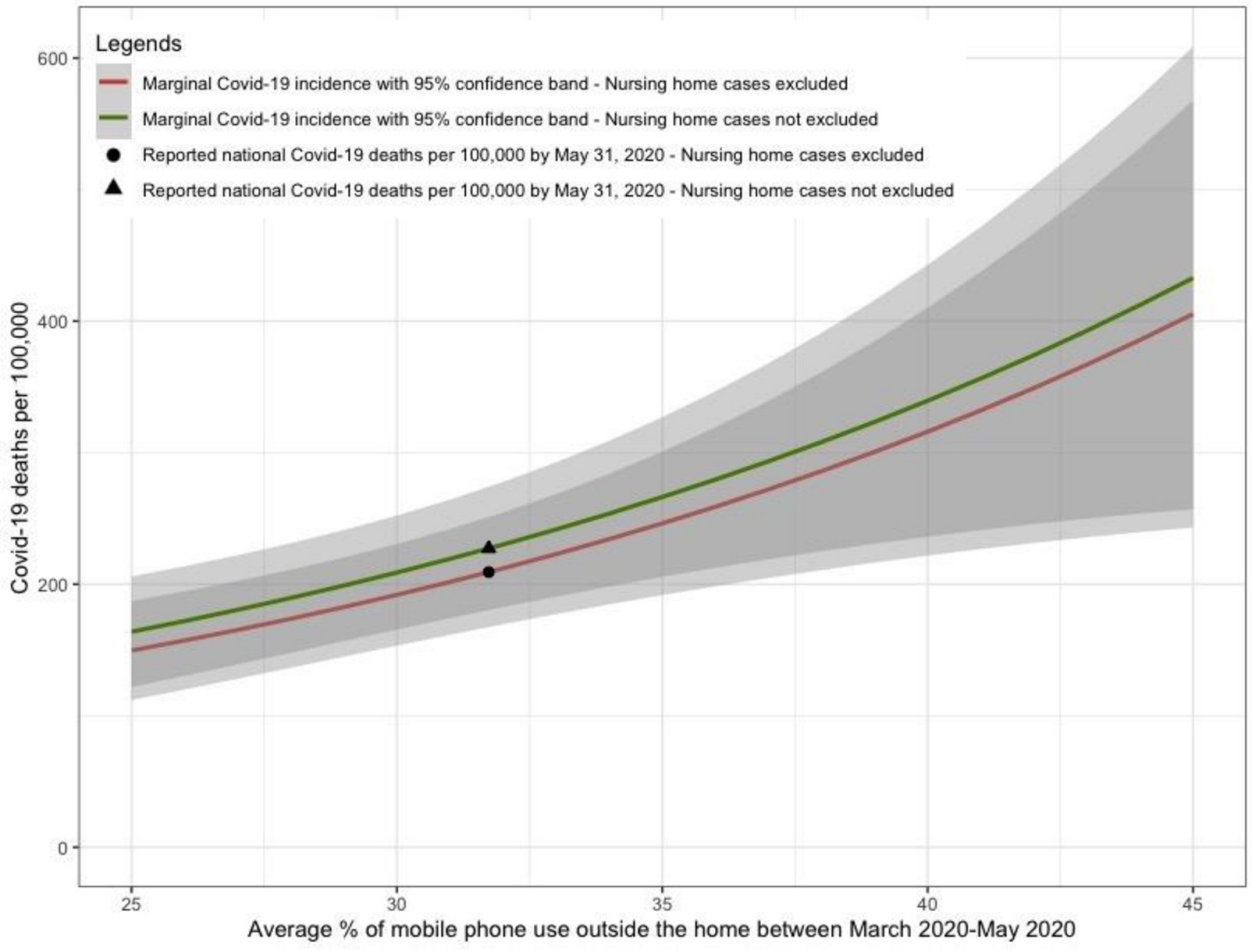
Estimated total confirmed COVID-19 cases (cases/100,000 people) by May 31^st^, 2020 versus average % of mobile devices leaving home (MDLH) between March 2020-May 2020.

Results from marginal effects at the mean analysis are presented in Fig 1. When social distancing was at the high end (25% of mobile devices leaving home between March 2020-May 2020), this corresponded with 163.84 total confirmed COVID-19 cases up to May 31^st^, 2020/100,000 people (95% CI: 121.81, 205.86) when nursing home COVID-19 cases were not excluded and 149.58 total confirmed COVID-19 cases up to May 31^st^, 2020/100,000 people (95% CI: 111.90, 187.26) when nursing home COVID-19 cases were excluded. In contrast, at the low end of social distancing (45% of mobile devices leaving home between March 2020-May 2020), these numbers were respectively 432.79 total confirmed COVID-19 cases up to May 31^st^/100,000 people (95% CI: 256.91, 608.66) and 405.38 total confirmed COVID-19 cases up to May 31^st^/100,000 people (95% CI: 243.14, 567.62) when nursing home COVID-19 cases were not excluded and when these cases were excluded.

## Discussion

In this nationwide study, we examined how excluding nursing home COVID-19 cases impacts the relationship between county-level social distancing behavior and COVID-19 cases in each US county during the early phase of the pandemic. A 1% increase in average % of mobile devices leaving home between March 2020-May 2020 was found to be significantly associated with a 5% increase in the number of total confirmed COVID-19 cases in a county regardless of whether nursing home COVID-19 cases were excluded from analyses. Accordingly, when we separated the impact of social distancing from other factors, predicted total COVID-19 confirmed cases up to May 31^st^, 2020/100,000 people were similar between analyses that did not exclude and those that did exclude nursing home COVID-19 cases throughout the range of social distancing values as measured through mobile phone use. Overall, little difference in the influence of social distancing behavior on COVID-19 cases was noted between analyses that did not exclude and those that excluded nursing home COVID-19 cases.

Given that social distancing behavior can vary within a state, there have been a number of studies that assessed the association between social distancing and COVID-19 at the county level throughout the US [8–12]. These studies tend to focus on the association between county-level social distancing behavior and COVID-19 growth rates with Gao et al., Cobb and Seale, Rubin et al., and Courtemanche et al. reporting that while social distancing generally prevents COVID-19 spread the magnitude of this association varied by county depending on how well individuals within a county adhered to social distancing measures [8–10,12]. Research by Banerjee and Nowak that looked at the relationship between county-level social distancing behavior and the natural log of COVID-19 cases observed much the same [11]. While all these studies demonstrate that the impact of social distancing behavior on COVID-19 can differ widely between counties, they do not consider nursing home COVID-19 cases when examining these associations.

Social distancing behavior in nursing homes is not necessarily reflective of the general population within a county as factors such as overcrowding and a large proportion of residents with dementia make adherence to social distancing measures particularly challenging in nursing homes [27]. A study by Algase et al. found that overcrowding in nursing homes is especially common in dining areas where it is not practical for masks to be worn during mealtimes [28]. In addition, around 48% of nursing home residents have some form of dementia which can make it difficult for them to understand why social distancing measures are needed during the pandemic as well as consistently practice these measures [29]. With these factors contributing to differing social distancing patterns in nursing homes, it is notable that we obtained comparable estimates of the influence of social distancing on COVID-19 when nursing home COVID-19 cases were not excluded and when they were excluded. Given that nursing home COVID-19 data can sometimes be delayed or misreported, this finding provides evidence that reasonably similar estimates of the influence of social distancing on preventing COVID-19 cases can be calculated if nursing home COVID-19 data is not available [30,31]. However, further research is warranted to determine if this same finding applies when determining the impact of social distancing on COVID-19 growth rates and mortality.

Our study has several limitations that need to be considered. Social distancing encompasses many aspects such as stay at home orders, sheltering in place, maintaining physical distance in public areas, and avoidance of large group gatherings [3,4]. While our study was only able to assess some aspects of social distancing, use of mobile phone data allows for a more quantitative approach to tracking social distancing that may be less prone to bias [32]. Another limitation is the inability to capture social distancing information on people without a mobile phone [17]. However, our study does include social distancing data on a large portion of the US population as 95% of US adults have a mobile phone with little difference in mobile phone ownership across race, age, education, income, and urban/rural residence [33].

## Conclusion

Findings from our study indicate that the approach of not excluding nursing home COVID-19 cases from total COVID-19 case counts has little impact when estimating the relationship that county-level social distancing has on preventing COVID-19 cases in the general US population. Building on this work, we note the need for future research examining whether not excluding nursing home COVID-19 cases also has minimal influence on the relationship between county-level social distancing and other measures of COVID-19 prevention such as disease growth rate and mortality.

## Supporting information

S1 Appendix Model coding. (DOCX)

S1 Table Additional information on model parameters and goodness of fit. (DOCX)

## References

[1] Centers for Disease Control and Prevention. Cases in the U.S. 2020 [Available from: https://www.cdc.gov/coronavirus/2019-ncov/cases-updates/cases-in-us.html.

[2] Centers for Disease Control and Prevention. Situation Summary 2020 [Available from: https://www.cdc.gov/coronavirus/2019-ncov/cases-updates/summary.html.

[3] Centers for Disease Control and Prevention. Social Distancing 2020 [Available from: https://www.cdc.gov/coronavirus/2019-ncov/prevent-getting-sick/social-distancing.html.

[4] Kaiser Family Foundation. Lifting Social Distancing Measures in America: State Actions & Metrics 2020 [Available from: https://www.kff.org/coronavirus-policy-watch/lifting-social-distancing-measures-in-america-state-actions-metrics/.

[5] Moreland A, Herlihy C, Tynan MA, Sunshine G, McCord RF, Hilton C, et al. Timing of state and territorial COVID-19 stay-at-home orders and changes in population movement—United States, March 1–May 31, 2020. Morbidity and Mortality Weekly Report. 2020;69(35):1198. doi: 10.15585/mmwr.mm6935a2 32881851 PMC7470456

[6] Centers for Disease Control and Prevention. Nursing Home Care 2021 [Available from: https://www.cdc.gov/nchs/fastats/nursing-home-care.htm.

[7] Abrams HR, Loomer L, Gandhi A, Grabowski DC. Characteristics of U.S. Nursing Homes with COVID-19 Cases. J Am Geriatr Soc. 2020;68(8):1653–6. doi: 10.1111/jgs.16661 32484912 PMC7300642

[8] Gao S, Rao J, Kang Y, Liang Y, Kruse J, Dopfer D, et al. Association of Mobile Phone Location Data Indications of Travel and Stay-at-Home Mandates With COVID-19 Infection Rates in the US. JAMA network open. 2020;3(9):e2020485-e. 10.1001/jamanetworkopen.2020.20485 PMC7489834 32897373

[9] Cobb JS, Seale MA. Examining the effect of social distancing on the compound growth rate of COVID-19 at the county level (United States) using statistical analyses and a random forest machine learning model. Public Health. 2020;185:27–9. doi: 10.1016/j.puhe.2020.04.016 32526559 PMC7186211

[10] Rubin D, Huang J, Fisher BT, Gasparrini A, Tam V, Song L, et al. Association of Social Distancing, Population Density, and Temperature With the Instantaneous Reproduction Number of SARS-CoV-2 in Counties Across the United States. JAMA Netw Open. 2020;3(7):e2016099. doi: 10.1001/jamanetworkopen.2020.16099 32701162 PMC7378754

[11] Banerjee T, Nayak A. U.S. county level analysis to determine If social distancing slowed the spread of COVID-19. Rev Panam Salud Publica. 2020;44:e90. doi: 10.26633/RPSP.2020.90 32636878 PMC7334824

[12] Courtemanche C, Garuccio J, Le A, Pinkston J, Yelowitz A. Strong Social Distancing Measures In The United States Reduced The COVID-19 Growth Rate: Study evaluates the impact of social distancing measures on the growth rate of confirmed COVID-19 cases across the United States. Health Affairs. 2020: doi: 10.1377/hlthaff. 2020.00608 10.1377/hlthaff.2020.00608 32407171

[13] Johns Hopkins Coronavirus Resource Center. Coronavirus COVID-19 Global Cases by the Center for Systems Science and Engineering (CSSE) at Johns Hopkins University 2020 [Available from: https://systems.jhu.edu.

[14] Centers for Medicare and Medicaid Services. COVID-19 Nursing Home Data 2020 [Available from: https://data.cms.gov/stories/s/COVID-19-Nursing-Home-Data/bkwz-xpvg. 26110197

[15] Environmental Systems Research Institute. ArcGIS Pro. 2.5 ed. Redlands, CA, 2020.

[16] Schuchat A. Public health response to the initiation and spread of pandemic COVID-19 in the United States, February 24–April 21, 2020. MMWR Morbidity and mortality weekly report. 2020;69. doi: 10.15585/mmwr.mm6918e2 32379733 PMC7737947

[17] SafeGraph. SafeGraph COVID-19 Data Consortium 2020 [Available from: https://www.safegraph.com/covid-19-data-consortium.

[18] Price GN, van Holm E. The Effect of Social Distancing On The Spread of Novel Coronavirus: Estimates From Linked State-Level Infection And American Time Use Survey Data. 2020.

[19] Ngonghala CN, Iboi E, Eikenberry S, Scotch M, MacIntyre CR, Bonds MH, et al. Mathematical assessment of the impact of non-pharmaceutical interventions on curtailing the 2019 novel Coronavirus. Mathematical Biosciences. 2020;325:108364. doi: 10.1016/j.mbs.2020.108364 32360770 PMC7252217

[20] Nayak A, Islam SJ, Mehta A, Ko Y-A, Patel SA, Goyal A, et al. Impact of Social Vulnerability on COVID-19 Incidence and Outcomes in the United States. medRxiv. 2020:2020.04.10.20060962. doi: 10.1101/2020.04.10.20060962 32511437 PMC7217093

[21] United States Census Bureau. County Population Totals: 2010–2019 2020 [Available from: https://www.census.gov/data/tables/time-series/demo/popest/2010s-counties-total.html.

[22] Centers for Disease Control and Prevention. CDC SVI 2018 Documentation 2020 [Available from: https://svi.cdc.gov/Documents/Data/2018_SVI_Data/SVI2018Documentation.pdf.

[23] Chen Y. New approaches for calculating Moran’s index of spatial autocorrelation. PLoS One. 2013;8(7):e68336. doi: 10.1371/journal.pone.0068336 23874592 PMC3709922

[24] StataCorp. Stata Statistical Software: Release 17. College Station, TX: StataCorp LLC; 2021.

[25] Williams R. Using Stata’s Margins Command to Estimate and Interpret Adjusted Predictions and Marginal Effects. The Stata Journal 2012;12(2):308–31.

[26] R Development Core Team. R: A language and environment for statistical computing 4.0 ed. Vienna, Austria R Foundation for Statistical Computing 2020.

[27] Brown KA, Jones A, Daneman N, Chan AK, Schwartz KL, Garber GE, et al. Association Between Nursing Home Crowding and COVID-19 Infection and Mortality in Ontario, Canada. JAMA Internal Medicine. 2021;181(2):229–36. doi: 10.1001/jamainternmed.2020.6466 33165560 PMC7653540

[28] Algase DL, Antonakos C, Beattie E, Beel-Bates C, Song JA. Estimates of crowding in long-term care: comparing two approaches. Herd. 2011;4(2):61–74. doi: 10.1177/193758671100400206 21465435

[29] Centers for Disease Control and Prevention. Alzheimer Disease 2021 [Available from: https://www.cdc.gov/nchs/fastats/alzheimers.htm.

[30] Chatterjee P, Kelly S, Qi M, Werner RM. Characteristics and Quality of US Nursing Homes Reporting Cases of Coronavirus Disease 2019 (COVID-19). JAMA Network Open. 2020;3(7):e2016930-e. 10.1001/jamanetworkopen.2020.16930 PMC8310566 32725243

[31] White EM, Wetle TF, Reddy A, Baier RR. Front-line Nursing Home Staff Experiences During the COVID-19 Pandemic. Journal of the American Medical Directors Association. 2021;2(1):199–203. doi: 10.1016/j.jamda.2020.11.022 33321076 PMC7685055

[32] Johnson TP, O’Rourke DP, Burris JE, Warnecke RB. An investigation of the effects of social desirability on the validity of self-reports of cancer screening behaviors. Medical care. 2005:565–73. doi: 10.1097/01.mlr.0000163648.26493.70 15908851

[33] Pew Research Center. Mobile fact sheet. Pew Research Center: Internet, Science & Tech. 2017.

