# Supplementary material for: Impact of excluding nursing home COVID-19 cases when assessing the relationship between county-level social distancing behavior and COVID-19 cases across the US during the early phase of the pandemic, February 2020-May 2020
Source: PLoS One. 2021 Nov 30;16(11):e0260151. doi: 10.1371/journal.pone.0260151 (PMC8631610; doi:10.1371/journal.pone.0260151)
Supplement: S1 Appendix — (DOCX) [file pone.0260151.s001.docx]

Variables used in mixed effects negative binomial models

| Variable Name | Description |
| --- | --- |
| menbreg | Variable to fit a mixed-effects negative binomial regression |
| rate5312020 | Rate of COVID-19 cases on May 31, 2020 |
| km2popden | Population density of a county per square kilometer |
| days1s5case531 | Days between the first confirmed case and May 31, 2020 |
| ep_pov | County percentage of population below the federal poverty line |
| ep_unemp | County percentage of population unemployed |
| ep_pci | County per-capita income |
| ep_nohsdp | County percentage of population older than 25 with no high school diploma |
| ep_age65 | County percentage of population 65 or older |
| ep_age17 | County percentage of population 17 or younger |
| ep_disabl | County percentage of civilian, non-institutionalized population who are disabled |
| ep_sngpnt | County percentage of population in a single parent household with one or more children younger than 18 |
| ep_minrty | County percentage of population who are a minority |
| ep_limeng | County percentage of population who speak English “less than well” |
| ep_munit | County percentage of housing composed of structures with 10 or more individual units |
| ep_mobile | County percentage of housing composed of mobile homes |
| ep_crowd | County percentage of housing units with more residents than rooms |
| ep_noveh | County percentage of households with no vehicles |
| ep_groupq | County percentage of population in institutionalized group housing |

Codes for mixed effects negative binomial models

- *menbreg rate5312020 km2popden days1stcase531 ep_pov ep_unemp ep_pci ep_nohsdp ep_age65 ep_age17 ep_disabl ep_sngpnt ep_minrty ep_limeng ep_munit ep_mobile ep_crowd ep_noveh ep_groupq sd_devleavinghome_feb sd_devleavinghome_345 _ || st_abbr:, irr*
- *menbreg RatesubstractNuHoConfirmed km2popden days1stcase531 ep_pov ep_unemp ep_pci ep_nohsdp ep_age65 ep_age17 ep_disabl ep_sngpnt ep_minrty ep_limeng ep_munit ep_mobile ep_crowd ep_noveh ep_groupq sd_devleavinghome_feb sd_devleavinghome_345 || st_abbr:, irr*
