# Supplementary material for: Impact of excluding nursing home COVID-19 cases when assessing the relationship between county-level social distancing behavior and COVID-19 cases across the US during the early phase of the pandemic, February 2020-May 2020
Source: PLoS One. 2021 Nov 30;16(11):e0260151. doi: 10.1371/journal.pone.0260151 (PMC8631610; doi:10.1371/journal.pone.0260151)
Supplement: S1 Table — (DOCX) [file pone.0260151.s002.docx]

|  | Mixed-effects negative binomial regression - nursing home COVID-19 cases included  IRR (95% CI) | Mixed-effects negative binomial regression - nursing home COVID-19 cases excluded  IRR (95% CI) |
| --- | --- | --- |
| population density (persons/sq km) | 1.00009 (1.00002, 1.00016) | 1.00011 (1.00004, 1.00018) |
| days between when the first confirmed COVID-19 case was reported in a county and May 31st, 2020 | 1.045 (1.042, 1.048) | 1.045 (1.042, 1.047) |
| % of population below poverty | 0.985 (0.974, 0.996) | 0.986 (0.975, 0.997) |
| unemployment rate | 1.016 (1.005, 1.028) | 1.016 (1.004, 1.027) |
| per-capita income | 1.000 (1.000, 1.000) | 1.000 (1.000, 1.000) |
| % of population >25 years with no high school diploma | 1.065 (1.050, 1.081) | 1.064 (1.048, 1.079) |
| % of population >65 years | 1.017 (1.001, 1.034) | 1.017 (1.001, 1.033) |
| % of population <17 years | 0.996 (0.974, 1.018) | 0.995 (0.974, 1.017) |
| % of civilian non-institutionalized population with a disability | 0.964 (0.949, 0.979) | 0.967 (0.952, 0.982) |
| % of population that is a single parent household with children <18 years | 1.044 (1.021, 1.066) | 1.046 (1.024, 1.069) |
| % of population that is a minority | 1.018 (1.014, 1.022) | 1.018 (1.014, 1.022) |
| , % of population >5 years who speak English “less than well” | 1.045 (1.017, 1.075) | 1.055 (1.027, 1.085) |
| % of housing that is a structure with >10 units | 1.004 (0.992, 1.015) | 1.001 (0.989, 1.012) |
| % of housing that is a mobile home | 1.006 (0.999, 1.013) | 1.005 (0.999, 1.012) |
| % of occupied housing units with more people than rooms | 0.971 (0.947, 0.994) | 0.976 (0.952, 1.000) |
| , % of households with no vehicles | 0.982 (0.967, 0.997) | 0.980 (0.965, 0.995) |
| % of population in institutionalized group quarters | 1.032 (1.021, 1.044) | 1.035 (1.024, 1.047) |
| sd_devleavinghome_feb | 0.975 (0.953, 0.997) | 0.974 (0.952, 0.996) |
| sd_devleavinghome_345 | 1.050 (1.024, 1.077) | 1.051 (1.025, 1.078) |
| Constant | 1.514 (0.556, 4.120) | 1.334 (0.494, 3.601) |
| Intra-level correlation coefficient - State | 0.402 (0.251, 0.646) | 0.382 (0.238, 0.611) |
| Conditional overdispersion Alpha | 1.006 (0.959, 1.056) | 1.003 (0.956, 1.052) |
| Log likelihood | -19669.297 | -19453.644 |
| Prob > Chi2 | 0.0000 | 0.0000 |
| LR test vs. negative binomial model | chibar2(01) = 488.65  Prob >= chibar2 = 0.0000 | chibar2(01) = 499.92  Prob >= chibar2 = 0.0000 |
